# Supplementary material for: Nonadiabatic Excited-State Dynamics with Quantum Monte Carlo-Trained Machine Learning: Azomethane as a Stringent Test
Source: J Chem Theory Comput. 2026 Sep 23;22(19):10180–91. doi: 10.1021/acs.jctc.6c01442 (PMC13671172; doi:10.1021/acs.jctc.6c01442)
Supplement: Supplementary file 1 [file ct6c01442_si_001.pdf]

Supporting Information: Nonadiabatic excited-state dynamics with quantum Monte Carlo-trained machine learning: azomethane as a stringent test

Alfonso Annarelli, Emiel Slootman, and Claudia Filippi*

MESA+ Institute for Nanotechnology, University of Twente, 7500 AE Enschede, The Netherlands

E-mail: c.filippi@utwente.nl

S1 Basis set and pseudopotential effects on vertical excitation energies

Table S1: CASSCF vertical excitation energies (eV) of *cis*-azomethane computed with different basis sets in all-electron (AE) and pseudopotential (BFD) calculations. The equilibrium geometry is optimized at the B3LYP/cc-pVTZ level using BFD pseudopotentials.

	$\Delta E_{\mathrm{CASSCF}}$ (eV)		
CAS(n,m) [no. dets]	BFD/aug-cc-pVDZ	AE/aug-cc-pVDZ	AE/aug-cc-pVTZ
(2,2) [4]	3.702	3.696	3.666
(6,4) [16]	3.685	3.682	3.653
(8,6) [225]	3.829	3.815	3.786

In Table S1, we assess the effect of basis set and pseudopotential on the CASSCF vertical excitation energies of *cis*-azomethane across the $(2e, 2o)$, $(6e, 4o)$, and $(8e, 6o)$ active spaces.

The latter extends the $(6e, 4o)$ active space by incorporating the σ-bonding and antibonding orbitals of the N−N bond. The excitation energies computed with the Burkatzki-Filippi-Dolg (BFD) pseudopotentials[1] show excellent agreement with the all-electron (AE) values, with a maximum discrepancy of about 0.01 eV (0.3 kcal/mol) for the $(8e, 6o)$ space. Increasing the basis set from aug-cc-pVDZ to aug-cc-pVTZ consistently lowers the excitation energies by approximately 0.03 eV (0.7 kcal/mol) for all active spaces.

A corresponding basis-set comparison at the VMC level, using fully optimized Jastrow-Slater wave functions constructed from the same active spaces, is reported in Table S2. At the VMC level, the excitation energies obtained with the two basis sets agree within statistical uncertainty.

Table S2: VMC vertical excitation energies (eV) of *cis*-azomethane, computed with fully optimized Jastrow-Slater wave functions built from different CAS expansions and basis sets. A two-body Jastrow factor is employed in all calculations. The equilibrium geometry is optimized at the B3LYP/cc-pVTZ level using BFD pseudopotentials. The statistical error on the last digit is reported in brackets.

	ΔE_{VMC} (eV)	
CAS(n,m)	BFD/aug-cc-pVDZ	BFD/aug-cc-pVTZ
(2,2)	3.59(1)	3.57(1)
(6,4)	3.62(1)	3.61(1)
(8,6)	3.63(1)	3.65(1)

S2 Basis set and Jastrow factor effects on forces

S2.1 CASSCF forces

For the CAS-based methods, the force benchmarks along the *cis*-isomerization and dissociation pathways reported in the main text employ the aug-cc-pVTZ basis set. By contrast, the *ab initio* CAS-based dynamics and reference data used to train the corresponding ML models are computed with the more affordable aug-cc-pVDZ basis set. We therefore quantify here

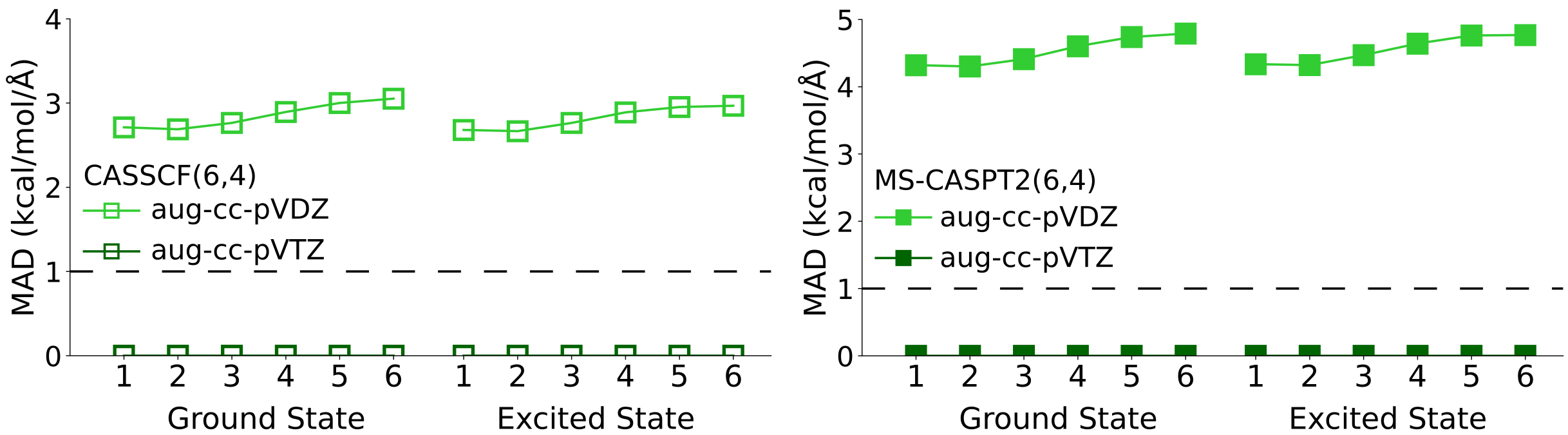

Figure S1: Effect of basis set on CASSCF(6,4) (left) and MS-CASPT2(6,4) (right) forces in kcal/mol/Å computed at geometries 1–6 along the *cis*-isomerization minimum energy path.

the basis-set dependence of the CASSCF and MS-CASPT2 forces along the isomerization pathway and then verify whether it affects the predicted nonadiabatic dynamics.

In Figure S1, we report the mean absolute deviation (MAD) of the CASSCF(6,4) and MS-CASPT2(6,4) forces computed with the aug-cc-pVDZ and aug-cc-pVTZ basis sets for geometries 1–6 along the *cis*-isomerization minimum energy path (see Figure 2 of the main text). The deviations are relatively uniform across the different geometries and are approximately 3 and 4.5 kcal/mol/Å for CASSCF and MS-CASPT2, respectively.

To assess whether these force differences affect the nonadiabatic dynamics, we train ML models on MS-CASPT2 data obtained with both basis sets. Figure S2 shows that the predicted excited-state populations for the *cis*-initiated photodynamics are essentially identical with aug-cc-pVDZ and aug-cc-pVTZ, indicating that the basis-set dependence of the CAS-based forces has a negligible impact on the predicted dynamics.

To further quantify this observation, we compute the average force root mean square (RMS),

$$\langle f \rangle(t) = \frac{1}{N_{\mathrm{trajs}}} \sum_{i}^{N_{\mathrm{trajs}}} \sqrt{\frac{1}{3N_{\mathrm{atom}}} \sum_{\alpha}^{N_{\mathrm{atom}}} \sum_{d=x,y,z} f_{i\alpha d}^{2}(t)}\,, \tag{S1}$$

over an ensemble of 1000 trajectories generated with ML models trained on data computed with the two basis sets, and report the results in Figure S3. The strong correlation over time between the force norms obtained with the two basis sets further confirms that the residual

basis-set dependence of the forces does not significantly affect the predicted dynamics.

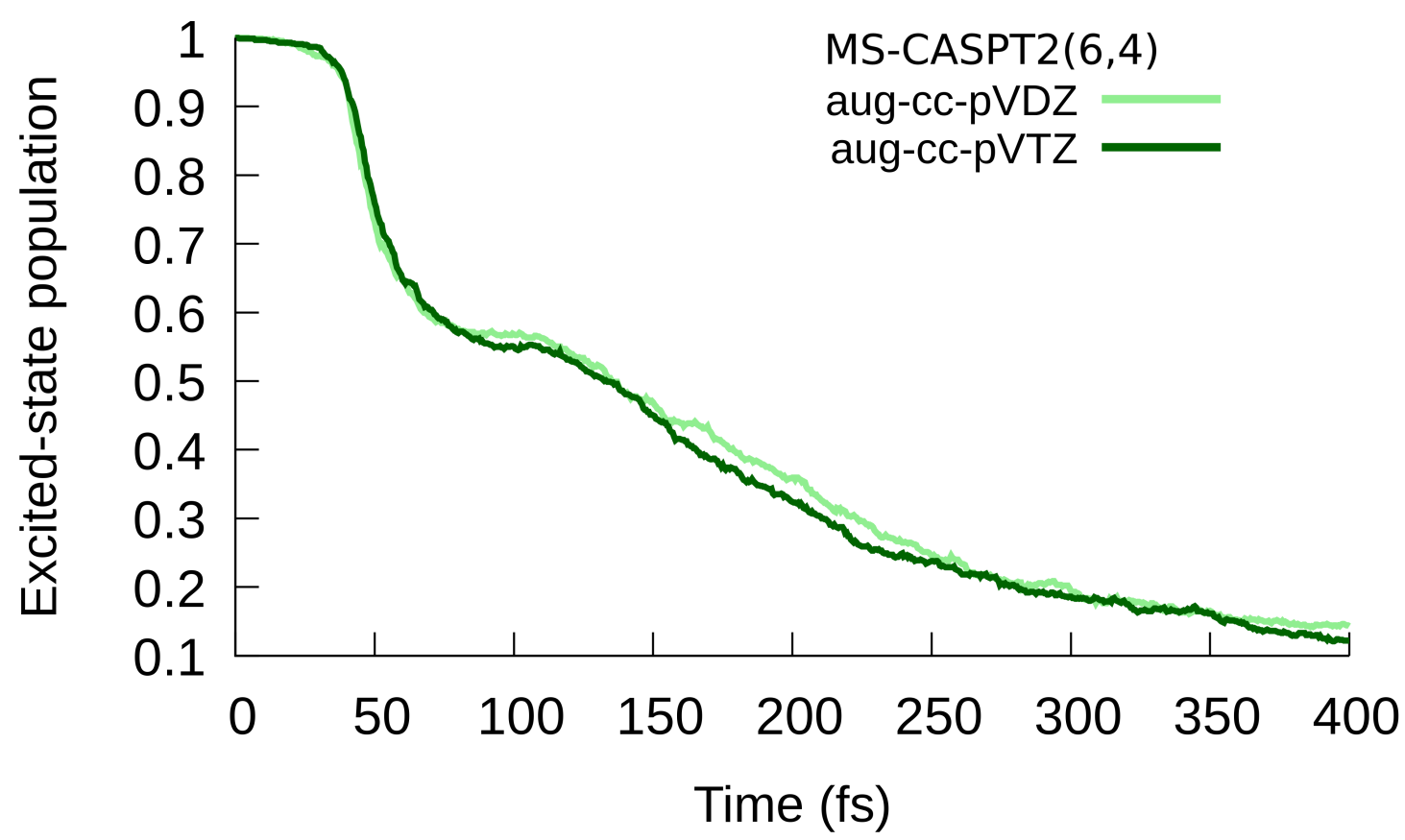

Figure S2: Excited-state population as a function of time for the *cis*-azomethane dynamics obtained with ML models trained on MS-CASPT2(6,4) data obtained with different basis sets. Each curve is averaged over an ensemble of 1000 trajectories.

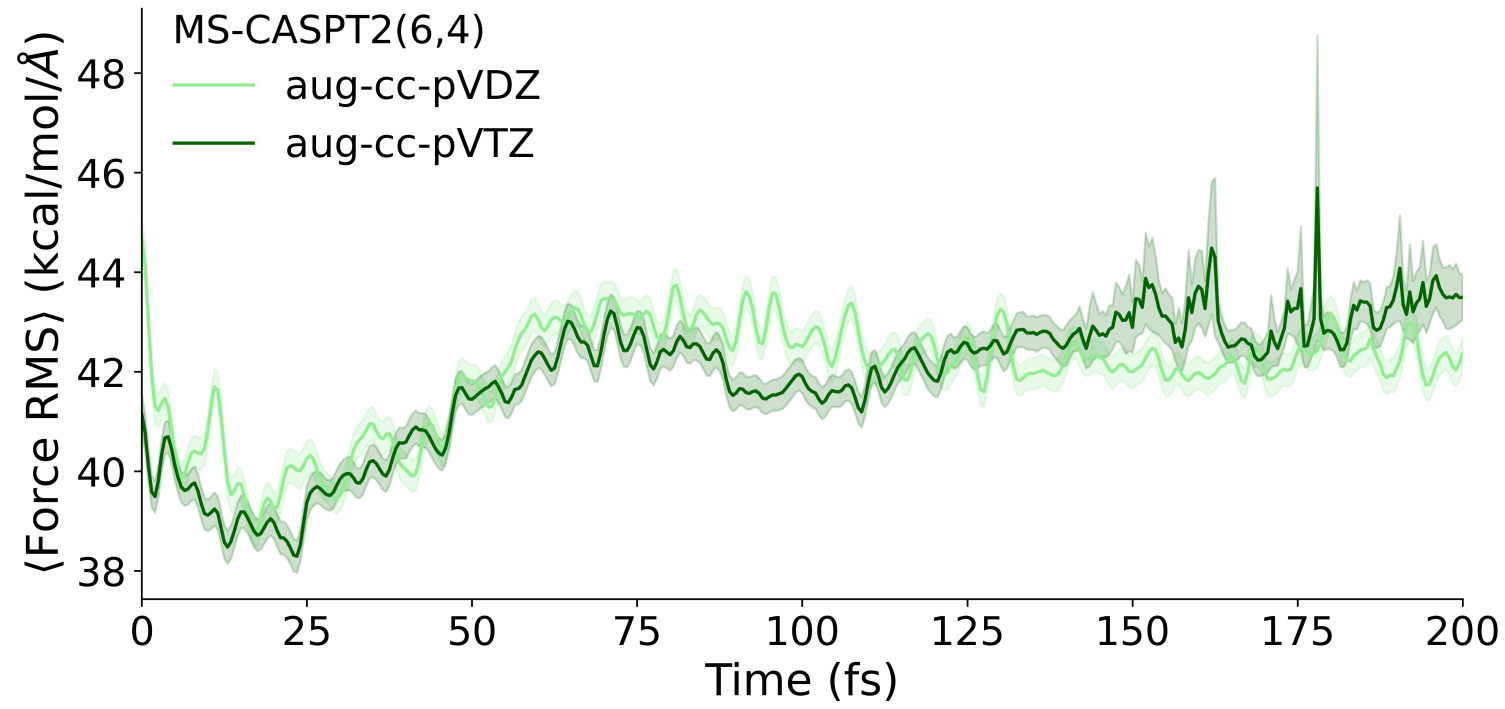

Figure S3: Average force RMS as a function of time for the cis-azomethane dynamics with ML models trained on MS-CASPT2(6,4) data obtained with different basis sets. Shaded regions indicate the statistical uncertainty estimated from 1000 trajectories.

Based on these considerations, the larger aug-cc-pVTZ basis set is adopted exclusively for the preliminary CASSCF and MS-CASPT2 benchmark calculations, whereas the more affordable aug-cc-pVDZ is preferred for the *ab initio* nonadiabatic molecular dynamics and the generation of the ML training data.

S2.2 VMC forces

The basis-set dependence of the VMC forces is assessed for geometries 1–4 using a CAS(6,4) determinantal expansion, as shown in Figure S4. The maximum MAD is 0.8 kcal/mol/Å, which is smaller than both the deviations between VMC and the other electronic structure methods discussed in the main text, whose lowest value is 1.2 kcal/mol/Å, and the test error of the ML-VMC force field (see Table S8). The aug-cc-pVDZ basis set therefore provides sufficient accuracy for generating the VMC training data used in the dynamics.

Similar cost-accuracy considerations also lead us to prefer a 2-body Jastrow factor over a 3-body variant: as shown in Figure S4, the force MAD between the two ranges from 0.9 to 1.1 kcal/mol/Å across the geometries 1–4 of the minimum energy path, a discrepancy that does not justify the higher computational cost required to generate the dataset.

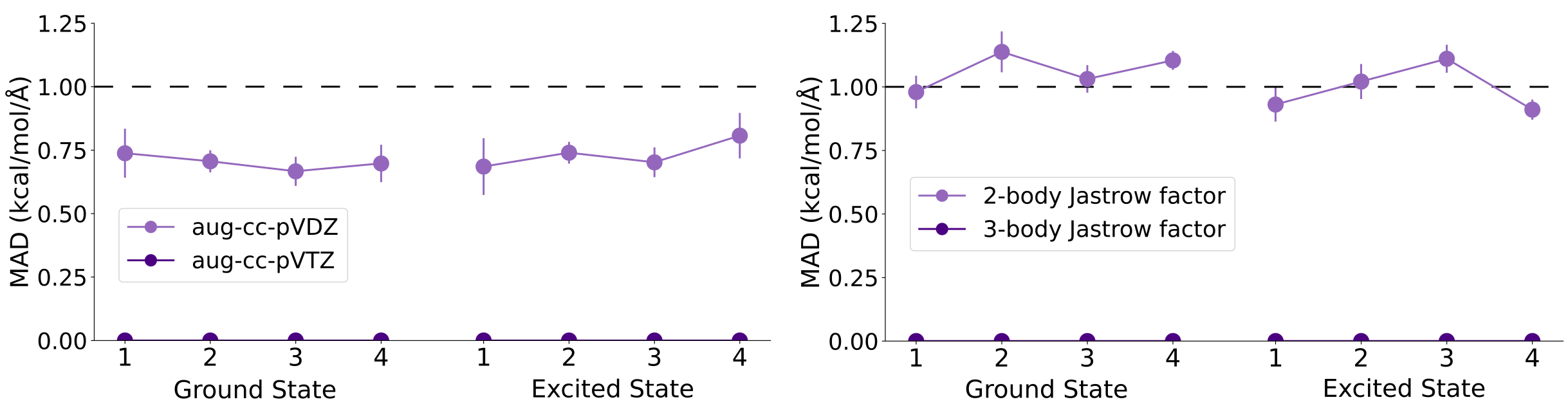

Figure S4: Effect of basis set and Jastrow factor on VMC forces (kcal/mol/Å) computed at geometries 1–4 along the *cis*-isomerization minimum energy path. Left: MAD between the aug-cc-pVDZ and aug-cc-pVTZ forces, both using a 2-body Jastrow factor. Right: MAD between forces obtained with 2-body and 3-body Jastrow factors, both using the aug-cc-pVDZ basis set. The forces are evaluated using a fully optimized Jastrow–Slater wave function with a CAS(6,4) determinant expansion.

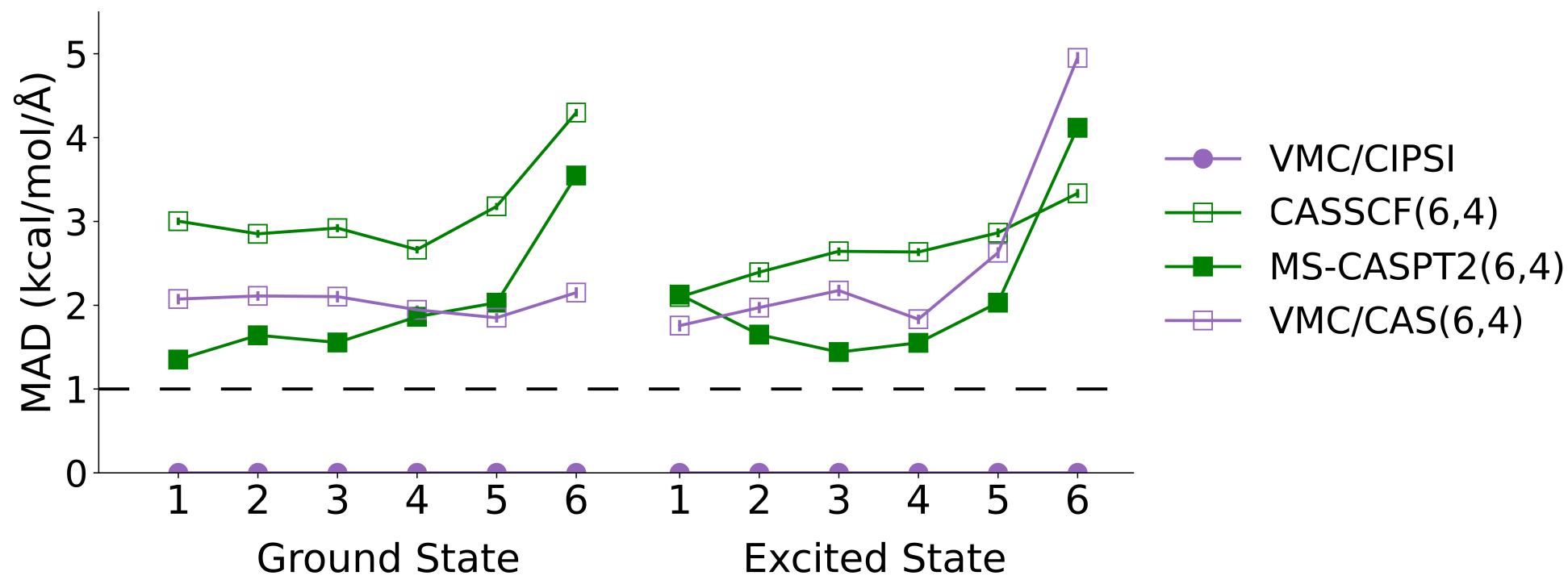

Figure S5: Force MADs between VMC calculations using CAS expansions and VMC/CIPSI benchmarks for the ground (left) and first excited state (right). VMC and CASSCF/CASPT2 calculations are performed with the aug-cc-pVDZ and aug-cc-pVTZ basis sets, respectively.

S3 VMC forces with CAS-based Jastrow-Slater wave functions

In Figure S5, we investigate the quality of the VMC forces obtained with a simple CAS(6,4) determinantal component in the fully optimized Jastrow-Slater wave function relative to the VMC/CIPSI reference. The corresponding MADs along the *cis*-isomerization path reveal that the VMC/CAS(6,4) forces are more accurate than the bare CASSCF ones. Yet, they are outperformed by the MS-CASPT2 forces across most configurations and exhibit larger deviations near the conical intersection (CI). As discussed in Section S5, the force differences observed near the conical intersection should be interpreted with caution, as they can partly reflect differences in the location of the crossing region between the methods.

Overall, the addition of a Jastrow factor to such a simple CAS expansion is insufficient to ensure the required, consistent accuracy. For the production dynamics, we thus employ the CIPSI-based VMC approach despite its higher computational cost.

S4 Effect of the CIPSI expansion size on VMC vertical excitation energies and forces

Table S3: VMC/CIPSI lowest vertical excitation energies (eV) of *cis*-azomethane as a function of the size of the determinantal expansion. The aug-cc-pVDZ basis set is employed. The equilibrium geometry is obtained at the B3LYP/cc-pVTZ level with BFD pseudopotentials. The bold entry corresponds to the expansion with a PT2 correction of −0.616 a.u. used in the main text.

N. dets	ΔE_{VMC}
117	3.582(6)
507	3.624(6)
1015	3.606(6)
**1200**	**3.581(4)**
2813	3.589(4)
5020	3.574(5)
10119	3.577(5)

The CIPSI expansions used in our Jastrow-Slater wave functions are generated by targeting the same PT2 energy correction (δE_{PT2}) of −0.616 a.u. for all states and geometries, leading to expansions with different numbers of determinants. We assess here whether this common threshold provides converged VMC/CIPSI vertical excitation energies and forces.

In Table S3, we report the VMC vertical excitation energies of *cis*-azomethane as a function of the number of determinants in the CIPSI expansion. The excitation energy is converged at approximately 1200 determinants, corresponding to the target PT2 correction of −0.616 a.u., and remains consistent with the values obtained using larger expansions within the statistical errors.

The convergence of the VMC/CIPSI forces is assessed by comparing a smaller expansion with $\delta E_{\mathrm{PT2}} = -0.650$ a.u., the production expansion with $\delta E_{\mathrm{PT2}} = -0.616$ a.u., and a larger expansion with $\delta E_{\mathrm{PT2}} = -0.582$ a.u. for geometries 1–6. For example, at geometry 1, these three thresholds correspond to expansions containing 702, 1129, and 1785 determinants, respectively. At geometry 6, the corresponding expansions contain 1146, 1716, and 3063

determinants. As shown in Figure S6, the force differences remain below 1 kcal/mol/Å across all configurations, except for configuration 2 when using the smallest expansion. We therefore conclude that the selected target δE_{PT2} provides sufficiently converged excitation energies and forces for the production calculations.

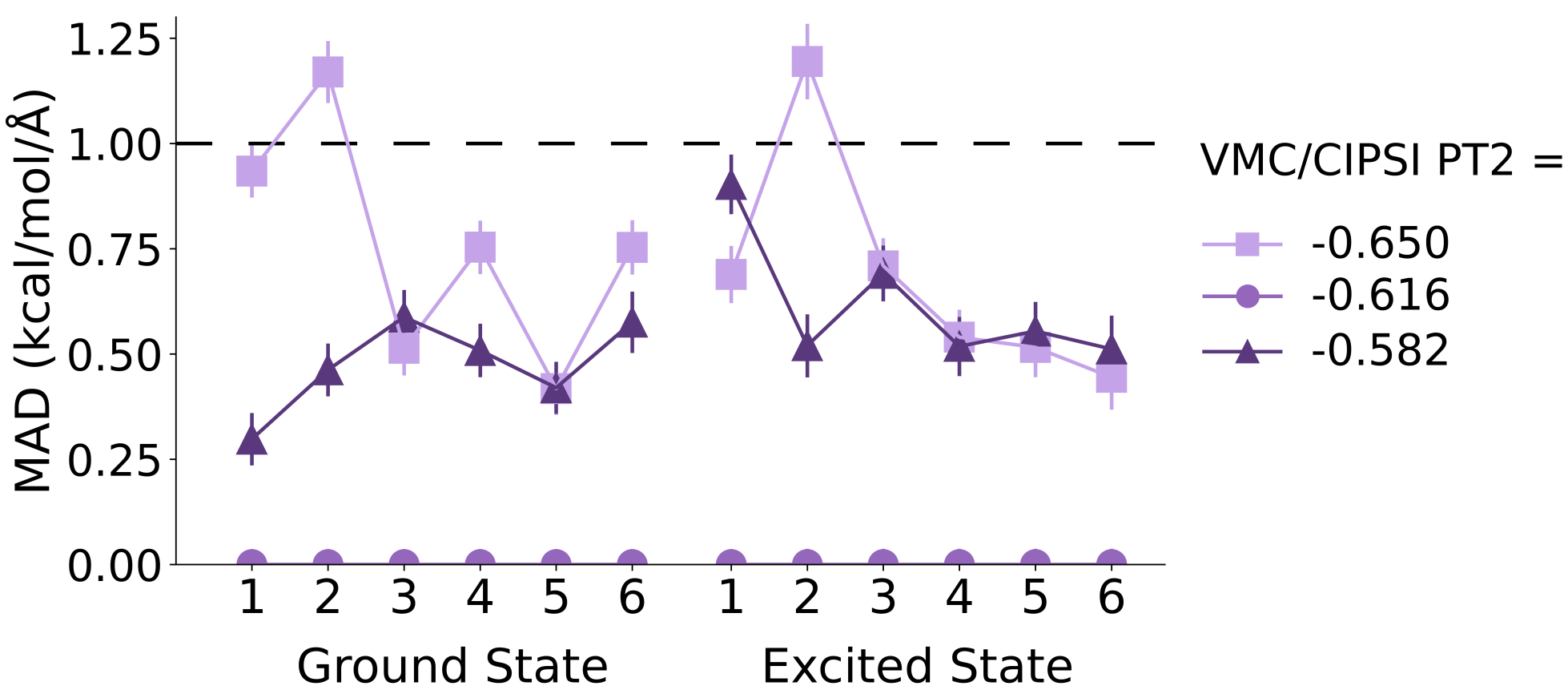

Figure S6: MADs of VMC/CIPSI forces (kcal/mol/Å) obtained with the production PT2 threshold of −0.616 a.u. relative to those obtained with smaller (−0.650 a.u.) and larger (−0.582 a.u.) determinantal expansions, for the ground state (left) and first excited state (right).

S5 Further analysis of the *ab initio* potential energy surfaces

Table S4 reports the *cis*-*trans* ground-state energy differences obtained with the different electronic structure methods. The VMC value lies between the exFCI and MRCI results, in line with the behavior observed for the vertical excitation energies discussed in the main text. The CASSCF values decrease as the active-space size is increased, while MS-CASPT2 further lowers the energy difference, underestimating both the VMC and exFCI references. We also report the ML-VMC prediction, which differs by 0.1 eV from the corresponding VMC/CIPSI reference. Further details on the ML models, including their predictive performance, are discussed in Section S9 and S10.

Table S4: *Cis-trans* ground-state energy differences (eV) computed at the optimal B3LYP/cc-pVTZ geometries. CASSCF and MS-CASPT2 calculations use the aug-cc-pVTZ basis set, while VMC and exFCI use aug-cc-pVDZ. The statistical error and extrapolation uncertainty on the last digit are indicated in parenthesis for the VMC and exFCI values, respectively.

Method	$\Delta E_{cis-trans}$
CASSCF(6,4)	0.44
CASSCF(12,10)	0.35
MS-CASPT2(6,4)	0.34
MS-CASPT2(12,10)	0.29
VMC/CIPSI	0.469(4)
ML-VMC/CIPSI	0.37
exFCI	0.433(9)
ADC(2)[a]	0.42
MRCI*PP[b]	0.48
L-PDFT[c]	0.40

[a]RI-ADC(2)/aug-cc-pVTZ from Ref. 2.
[b]MRCI(6,4)*PP/6-31G* from Ref. 2.
[c]L(2)-tPBE(6,4)/6-31G* from Ref. 3.

For the geometries along the CASSCF(6,4) *cis*-isomerization path used in Section 4.2 of the main text, we report the energies at different electronic structure theory levels in Figure S7. We find that all methods predict a closing of the energy gap as the system approaches the region with ∠CNNC $\sim 90°$, suggesting a qualitatively consistent location of the conical intersection.

Figure S7 shows also the energy profiles along the C–N dissociation path analyzed in Section 4.2. Interestingly, the CASSCF(12,10) ground-state energy decreases markedly relative to the other methods as the molecule approaches the dissociated geometries (10 and 11). This trend is consistent with the higher dissociation rate observed with CASSCF(12,10) in the dynamics.

To further characterize the region of the S_1/S_0 crossing, we compare the minimum-energy conical intersections (MECIs) obtained with the different CAS-based methods considered here. In Table S5, we report selected internal coordinates of the corresponding MECIs. The ∠CNNC is close to 90° for all methods, while the C–N bond lengths show a

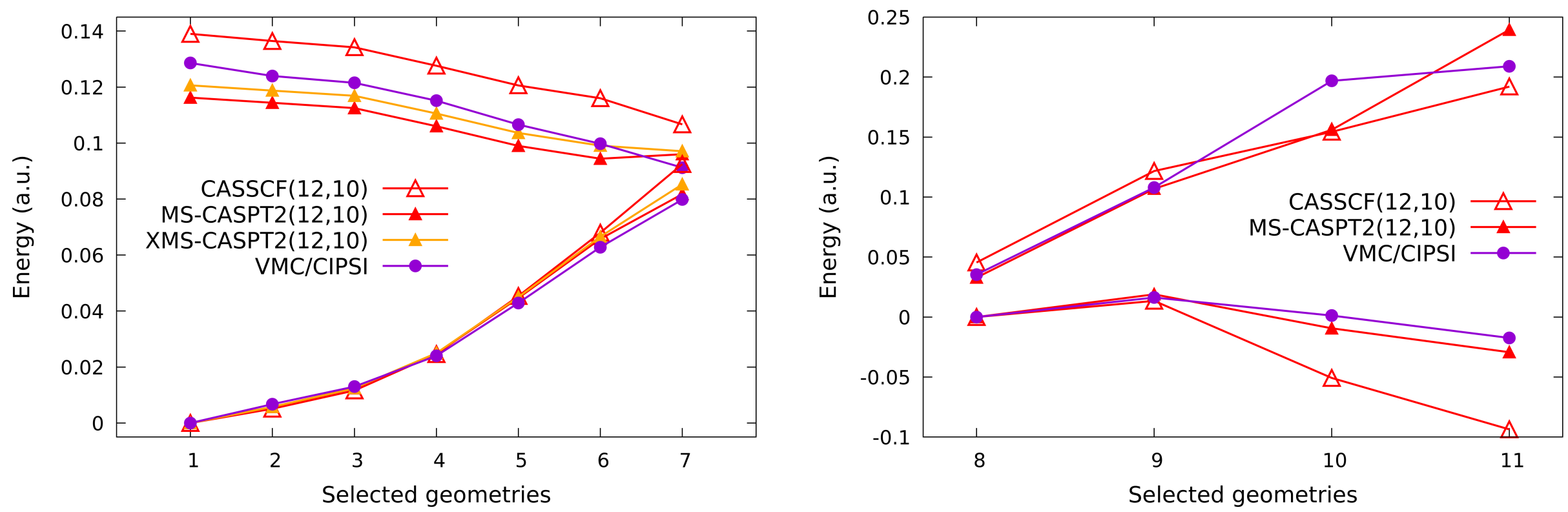

Figure S7: Ground- and excited-state energies computed along the CASSCF(6,4) *cis*-isomerization minimum energy path (left) and the C–N dissociation path (right). For each method, the energies are shifted such that the ground-state energies at geometry 1 (left) and 8 (right) are set to zero. CAS-based methods use aug-cc-pVTZ.

larger method dependence, with CASSCF(12,10) yielding the most stretched C–N bonds. A conical-intersection optimization is not available at the VMC level in our present implementation. Nevertheless, along the same minimum-energy path, VMC/CIPSI shows a closing of the S_1-S_0 energy gap as the ∠CNNC dihedral approaches 90° like the CAS-based methods (Figure S7).

Table S5: Selected internal coordinates of the minimum-energy conical intersections (MECIs) obtained at different levels of theory with the aug-cc-pVDZ basis set. Bond distances are in Å and dihedral angles in degrees.

Internal coordinates	CASSCF(6,4)	CASSCF(12,10)	MS-CASPT2(12,10)	XMS-CASPT2(12,10)
N_1–N_2	1.275	1.281	1.267	1.272
N_1–C_1	1.438	1.478	1.443	1.445
N_2–C_2	1.464	1.542	1.499	1.487
C_1–H_1	1.085	1.085	1.098	1.095
$\angle C_2N_2N_1C_1$	-93.69	-93.75	-91.75	-92.13

To assess how the differences in the MECI geometries affect the forces, we compare the MS-CASPT2 and XMS-CASPT2 forces computed at the CASSCF(6,4) MECI with those computed at their respective MECIs. We report the corresponding mean absolute deviations in Table S6. These deviations are smaller than the MADs between different methods at

geometry 7 near the conical intersection along the CASSCF(6,4) minimum-energy path, which reach up to 14 kcal/mol/Å, but are still substantial. This indicates that the large force differences at geometry 7 may partly reflect differences in MECI locations, in addition to differences in the description of the near-degenerate states.

Table S6: Mean absolute deviations (MADs, kcal/mol/Å) between the (X)MS-CASPT2(12,10) forces computed at the CASSCF(6,4) MECI and those computed at their respective MECIs.

Method	MAD (S_0)	MAD (S_1)
MS-CASPT2(12,10)	6.62	9.78
XMS-CASPT2(12,10)	7.17	5.17

S6 Forces on single-dissociated geometry

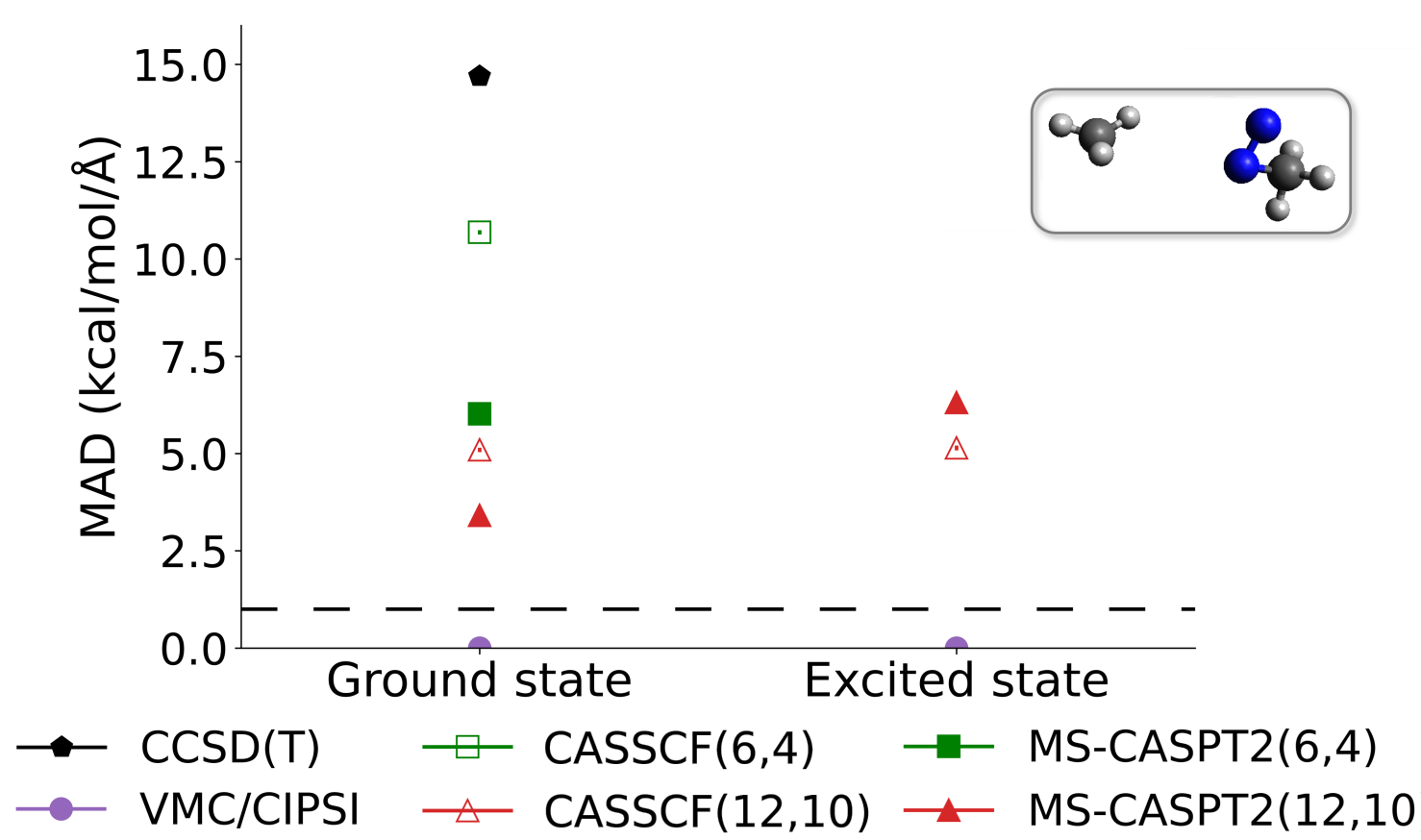

Figure S8: Force MADs (kcal/mol/Å) computed for a single-dissociated geometry relative to VMC/CIPSI values for the ground (left) and the first excited state (right). CC calculations use the cc-pVQZ basis set and CAS-based methods use aug-cc-pVTZ.

Figure S8 reports the force MADs relative to VMC/CIPSI for the different electronic structure methods at a single-dissociated geometry, corresponding to $CH_3N_2^{\bullet} + CH_3^{\bullet}$. As in the case of the double-dissociation pathway, coupled cluster performs poorly due to its single-reference nature. The MS-CASPT2 ground-state forces exhibit the closest agreement

with the VMC/CIPSI reference. For the excited state, CASSCF(12,10) correctly recovers the dominant $n_- \rightarrow \pi^*$ excitation localized on the $CH_3N_2{}^{\bullet}$ fragment. Nevertheless, despite this correct electronic character, both CASSCF and MS-CASPT2 excited-state forces still deviate significantly from the VMC/CIPSI reference, with MADs exceeding 5 kcal/mol/Å.

S7 Comparison between MS- and XMS-CASPT2 forces and dynamics

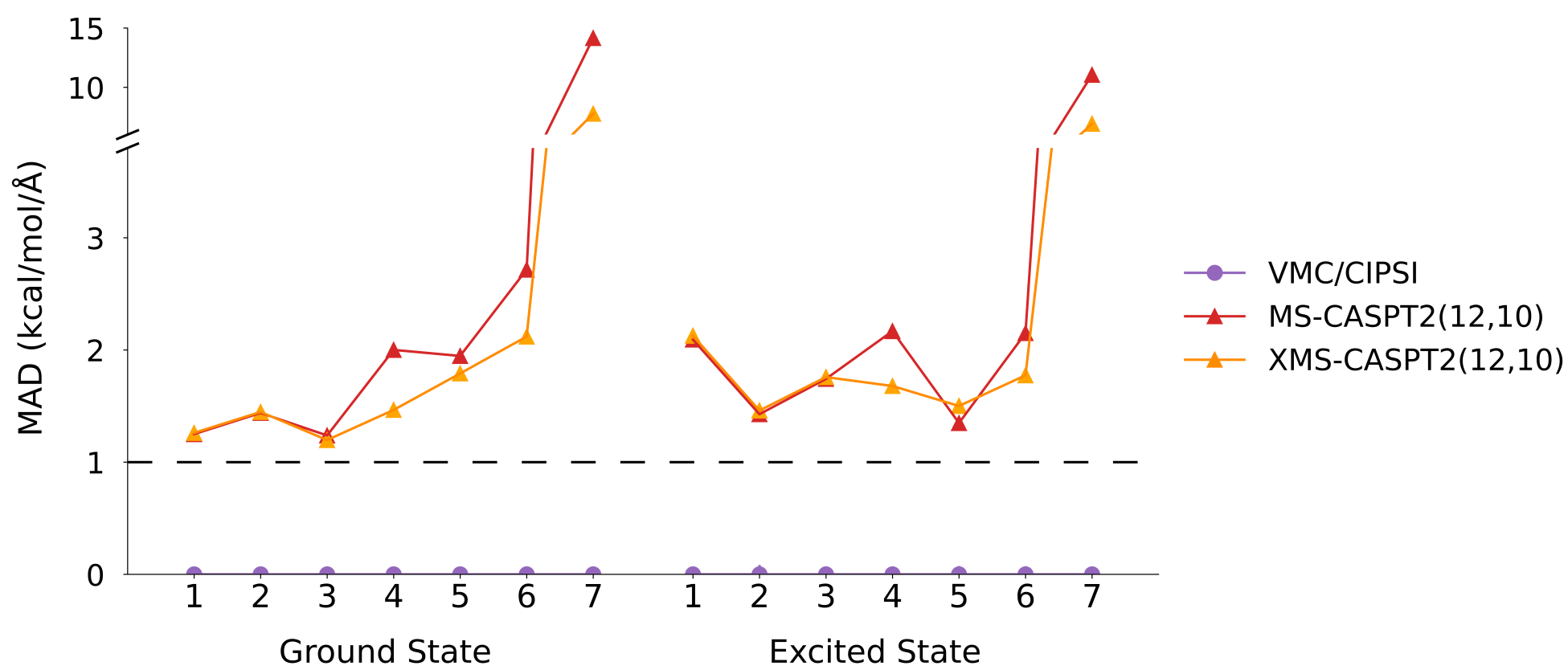

Figure S9: Force MADs (kcal/mol/Å) for MS- and XMS-CASPT2 relative to VMC/CIPSI reference for the ground (left) and first excited state (right). VMC and CASPT2 calculations are performed with the aug-cc-pVDZ and aug-cc-pVTZ basis sets, respectively.

To assess the effect of the extended multi-state formulation, we compare MS-CASPT2 and XMS-CASPT2 for the forces along the cis-isomerization pathway and for the resulting nonadiabatic dynamics. As shown in Figure S9, the two methods give overall similar force deviations relative to VMC/CIPSI along the rotation pathway. Near the degeneracy region, XMS-CASPT2 gives somewhat smaller deviations, reducing the maximum MAD from about 14 to below 8 kcal/mol/Å for both states.

We further compare the two methods through nonadiabatic dynamics. To this end, an ML model is trained on the same 2320-configuration dataset at the XMS-CASPT2 level and is subsequently used to run 1000 surface-hopping trajectories for the *cis*-isomer photodynamics. Figure S10 shows that the excited-state populations obtained with the MS- and XMS-CASPT2 ML models are very similar. Therefore, for this system, XMS-CASPT2 does not lead to substantial changes in the dynamics and MS-CASPT2 is retained for the discussion in the main text.

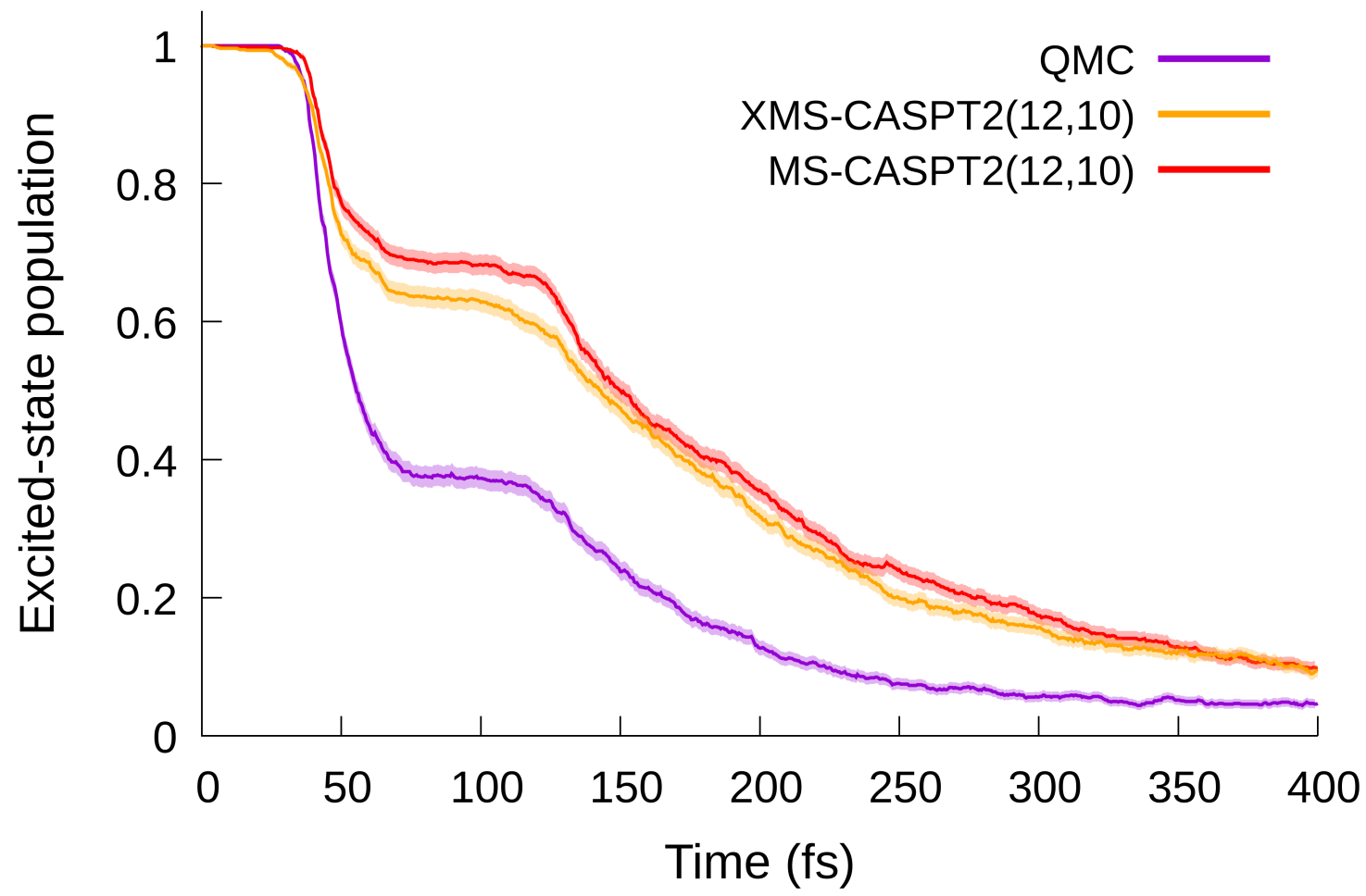

Figure S10: Time evolution of the excited-state population from 1000 ML trajectories using XMS-CASPT2, compared with the corresponding MS-CASPT2- and QMC-based ML dynamics. All trajectories are initiated from the *cis* isomer.

S8 Effect of the coupling approximation on S_1 population decay

To verify the robustness of the trajectory propagation with respect to the treatment of nonadiabatic couplings, Figure S11 compares the overlap-based approach with the curvature-driven approximation used to estimate the time-derivative coupling terms in the nonadiabatic dynamics. For both CASSCF and MS-CASPT2, the S_1 population decay profiles obtained from 100 *ab initio* trajectories are qualitatively similar for the two coupling schemes. This agreement indicates that the population dynamics is only weakly affected by the coupling approximation and supports the use of curvature-driven trajectory surface hopping (κTSH) for the dynamics reported in the main text.

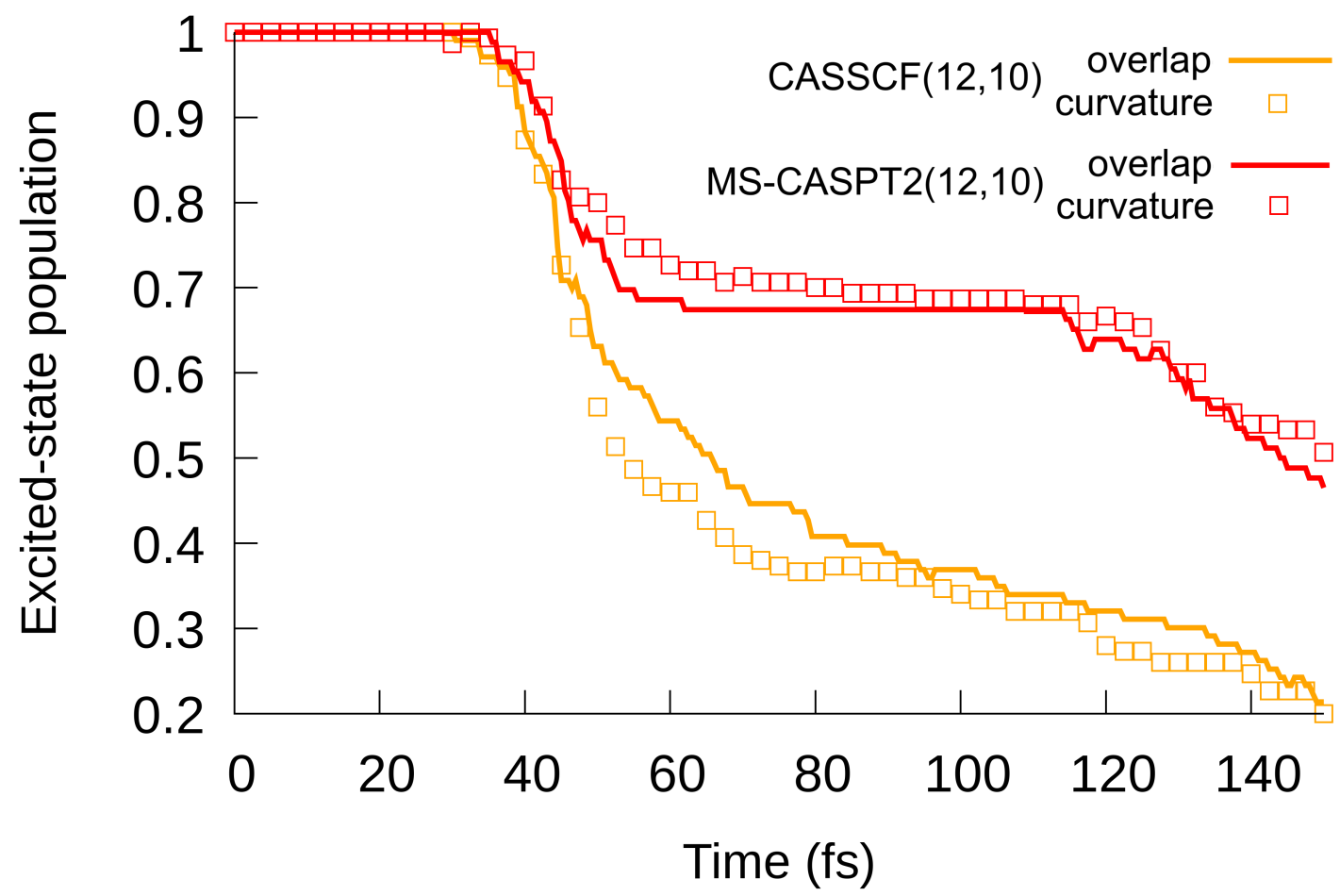

Figure S11: Time evolution of the excited-state populations for 100 *ab initio* trajectories initialized in the S_1 state of the *cis* isomer. The overlap-based approach is compared to the curvature-driven approximation used in κTSH, showing good agreement for both CASSCF(12,10) and MS-CASPT2(12,10).

S9 Neural network training details and adaptive sampling convergence

The models used in this work are message-passing graph neural networks (NN) based on the PaiNN architecture.[4] The interaction layers iteratively update the atomic environments to build the molecular representation, while a sequence of dense readout layers subsequently yields the energies of the two electronic states. Forces are computed as analytical gradients of these energies with respect to the atomic coordinates. The reference dataset is randomly partitioned into training, validation, and test sets using a 7:1:2 ratio. Training minimizes a mean squared error (MSE) loss function, computed as a weighted sum of the energy and force contributions. To improve numerical stability during training, the mean energy of the training set is subtracted from the input reference energies. Additionally, the learning rate is dynamically adjusted throughout the training process: it is multiplied by a decay factor (between 0 and 1) whenever the validation loss fails to improve for a specified number of consecutive epochs, referred to as the learning rate decay patience.

To generate a representative dataset of the dynamics, an adaptive sampling procedure, also termed active learning, is carried out at the CASSCF reference level, based on previously described methodologies.[5,6] The sampling is driven by excited-state dynamics using two independently trained NNs initialized from the same dataset. The two models, NN1 and NN2, share the architectural parameters summarized in Table S7, and the training procedures differ only in the initial learning rates and energy-to-force loss-weight ratios. At each iteration, the "lead" model (NN1) propagates 100 surface-hopping trajectories, while energy and force discrepancies with the "advisor" model (NN2) are evaluated at every encountered geometry. Configurations that exceed predefined uncertainty thresholds are added to the training set; individual trajectories are terminated either after two consecutive threshold failures or upon reaching 200 fs. The NNs are then retrained from scratch for the next iteration without using the previous weights as a starting guess. The energy and force thresholds are progressively reduced by a factor of 0.9 at each iteration until reaching minimum values of 0.005 Ha and 0.005 Ha/Bohr (approximately 3 kcal/mol and 6 kcal/mol/Å), respectively, after which they are kept constant.

Table S7: Main hyperparameters to construct the two NNs for adaptive sampling.

Property	NN1	NN2
Number of interaction layers	2	2
Number of readout layers	3	3
Energy/forces loss weight	0.5/1.0	0.6/0.9
Batch size	10	10
Learning rate (lr)	$1 \cdot 10^{-3}$	$9 \cdot 10^{-4}$
Decay factor of lr	0.95	0.95
lr decay patience (epochs)	500	500
Basis function	Gaussian radial BF	Gaussian radial BF

We start from a 50-configuration dataset sampled from a Wigner distribution at the *cis* geometry and perform the active learning at the CASSCF(6,4) level. This procedure samples densely the region close to the conical intersection, characterized by $|\angle \mathrm{CNNC}| \sim 90°$, as shown in Figure S12. To assess the convergence of the adaptive sampling, we monitor not only the distribution of geometries in the dataset but also the ML-predicted excited-state

population. With 752 geometries, the ML-predicted excited-state population converges to the *ab initio* reference and this dataset is then used as a starting point for a subsequent active-learning procedure at the CASSCF(12,10) level.

For the CASSCF(12,10) case shown in Figure S12, the algorithm again focuses on the conical-intersection region but also selects distorted geometries with elongated C–N bonds. Specifically, 9% of the collected configurations feature a C–N bond longer than 1.8 Å (compared with the equilibrium value of 1.47 Å), leading to a final dataset of 2320 points.

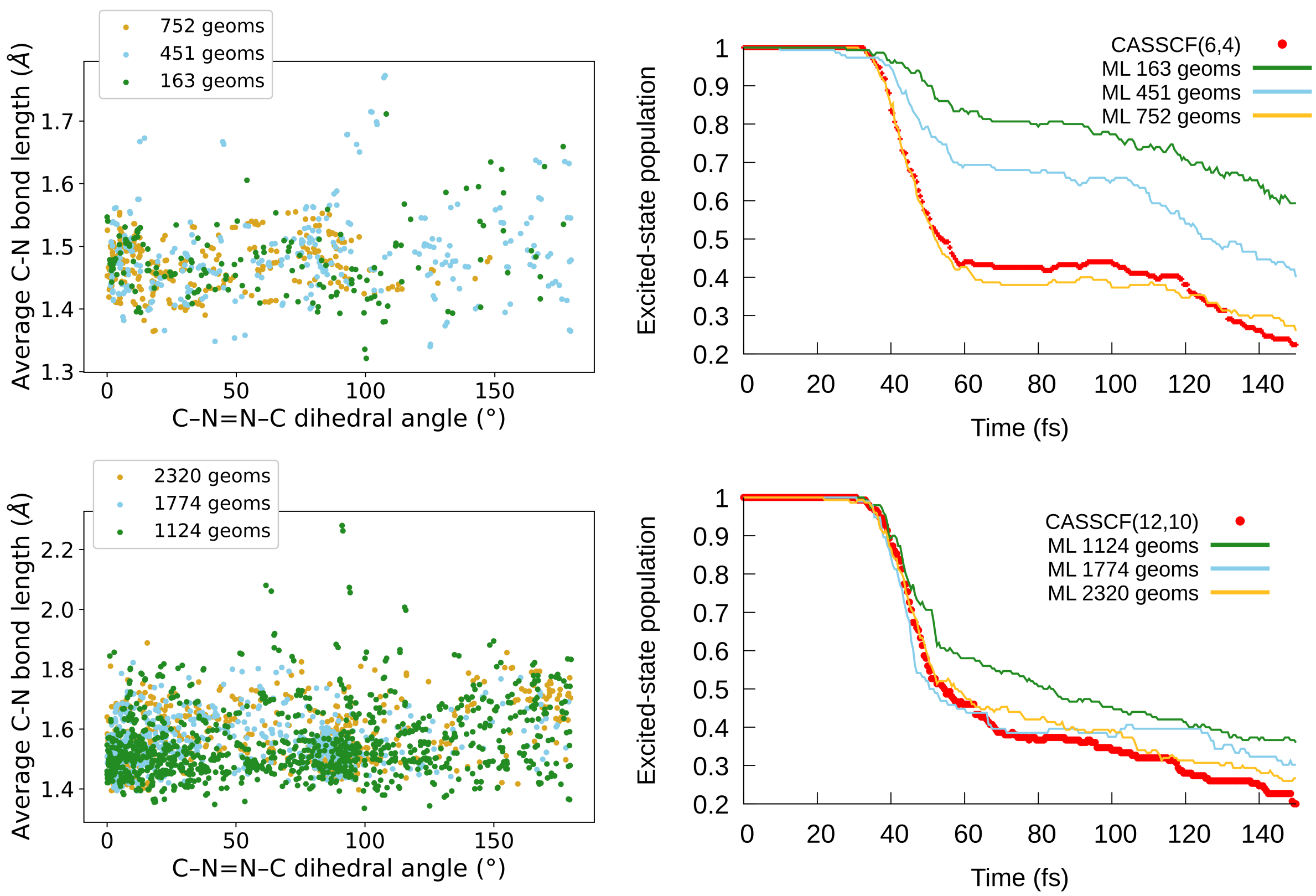

Figure S12: Adaptive sampling based on CASSCF dynamics with the $(6e, 4o)$ active space (top row) and the $(12e, 10o)$ active space (bottom row). Left panels: distribution of sampled data-set configurations as a function of the average C–N bond length and the C$-$N=N$-$C dihedral angle, tracked across different active-learning iterations. Right panels: convergence of the ML-predicted excited-state population toward the *ab initio* reference as a function of the dataset size. Both the *ab initio* reference and the ML populations are computed using 150 trajectories.

Figure S13 provides the RMS training and validation losses for the three main models

employed in the main text. Training is stopped when the validation loss no longer improves for 1500 consecutive epochs. In addition, Table S8 summarizes the test errors for the energies and forces of all ML models, computed on the respective test subsets. The errors are defined as the mean absolute error (MAE) of the ML predictions against the corresponding *ab initio* reference values, averaged over the two electronic states, and provide an estimate of the model fitting error. Interestingly, CASSCF(12,10) data are found to be particularly challenging for the ML model to learn compared to other *ab initio* methods. Differences between models smaller than these test errors are not meaningful, as they can be attributed to fitting noise rather than real discrepancies in the *ab initio* methods.

Table S8: Test errors of the SPaiNN models (in kcal/mol for energies and kcal/mol/Å for forces).

Method	Dataset size	MAE (energy)	MAE (forces)
CASSCF(6,4)	752	0.67	1.69
CASSCF(12,10)	2320	1.87	3.64
MS-CASPT2(12,10)	2320	0.53	2.26
XMS-CASPT2(12,10)	2320	0.50	2.06
VMC/CIPSI	2320	1.13	2.58

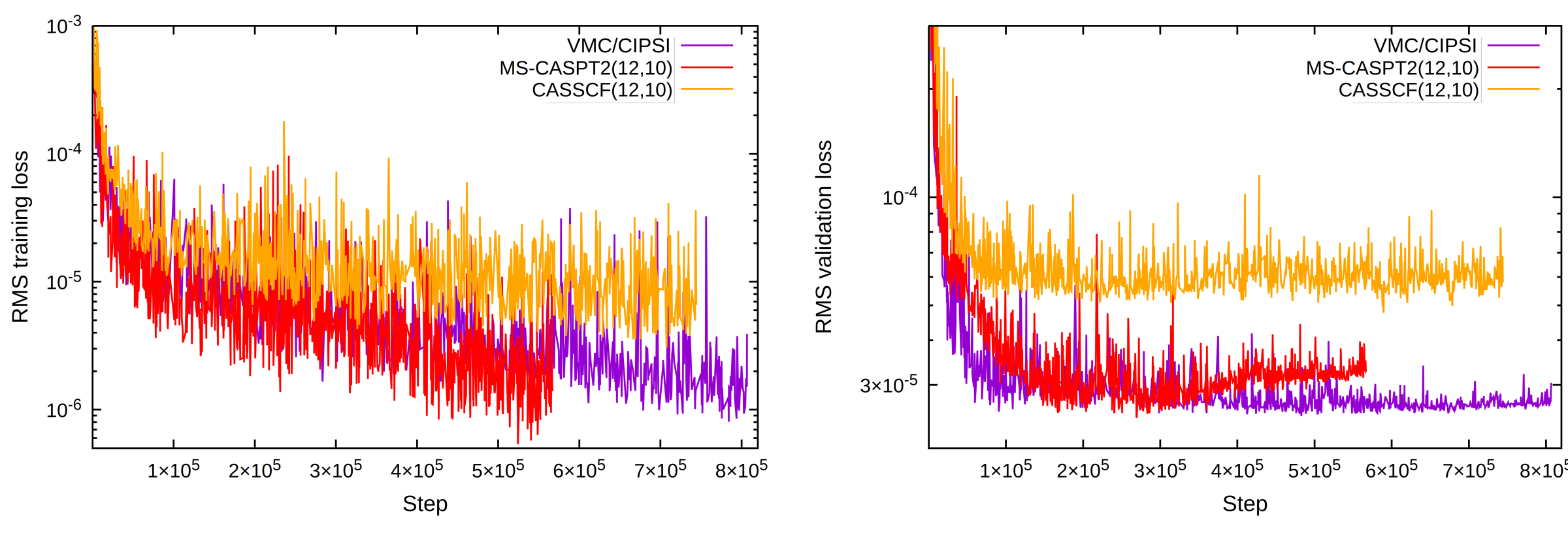

Figure S13: Training (left) and validation (right) loss curves for the ML models.

S10 Further tests of ML model performance

We first assess in Table S9 the performance of the three ML models used in the main text for predicting vertical excitation energies at the *cis* and *trans* geometries. Here, the *ab initio* CASSCF and CASPT2 excitation energies are computed using the aug-cc-pVDZ basis set, consistent with the level of theory used for model training. Both the VMC/CIPSI and MS-CASPT2 models show good agreement with their reference values, with deviations within 0.05 eV (1.2 kcal/mol). The CASSCF model exhibits larger deviations, while consistently predicting higher excitation energies than the other two models.

Table S9: Vertical excitation energies (eV) of *cis*- and *trans*-azomethane at the optimal B3LYP/cc-pVTZ geometries, computed with the reference electronic structure methods and the corresponding ML models. All *ab initio* calculations use the aug-cc-pVDZ basis set. The statistical error on the last digit are indicated in parenthesis.

Method	ΔE_{cis}	ΔE_{trans}
CASSCF(12,10)	4.00	4.20
ML-CASSCF(12,10)	3.89	3.98
MS-CASPT2(12,10)	3.45	3.68
ML-MS-CASPT2(12,10)	3.50	3.65
VMC/CIPSI	3.581(4)	3.769(4)
ML-VMC/CIPSI	3.634	3.754

A similar pattern is observed for the ML force benchmark. Figure S14 reports the MADs between the ML forces and their corresponding *ab initio* reference values along the *cis*-isomerization path. Both the ML-VMC and ML-MS-CASPT2 models yield accurate forces along the path, with deviations below 2 kcal/mol/Å from the reference, except near the conical intersection where the agreement deteriorates. The ML-CASSCF predictions generally show larger deviations than the other models, although they remain below 4 kcal/mol/Å, consistent with the model's test error reported in Table S8.

The degradation in neural-network performance near the conical intersection is somewhat expected, as NN potentials are smooth functions, while the adiabatic potential energy surfaces are singular at the intersection.[5] Nevertheless, as shown in the inset of Figure 5 for

MS-CASPT2 and in Figure S12 for CASSCF, these inaccuracies do not significantly affect the ML prediction of the excited-state population. This can be attributed to the stochastic hopping occurring over a relatively broad region around the CI, without ever reaching the CI seam itself.[7]

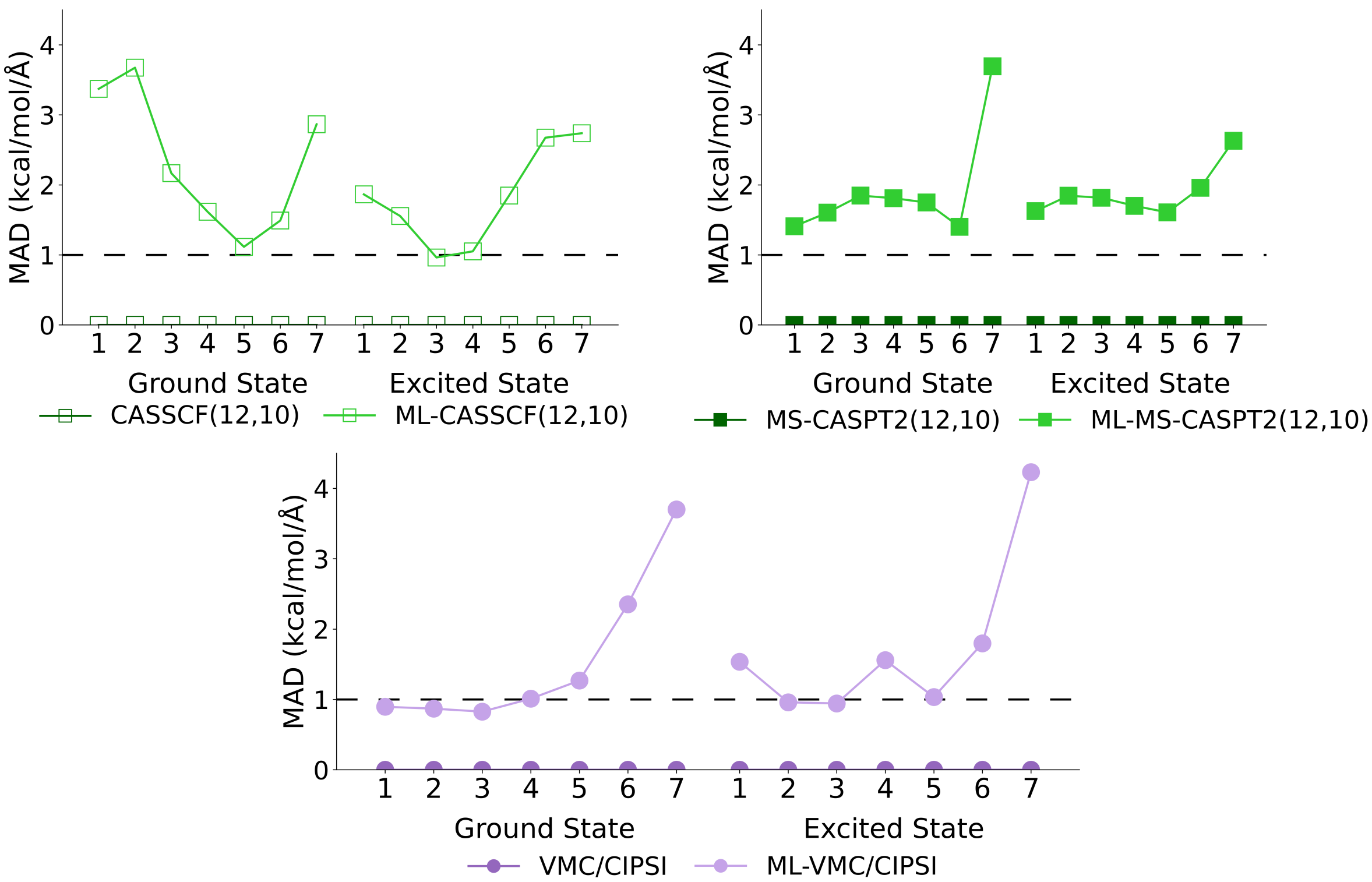

Figure S14: Force mean absolute deviations (MAD, kcal/mol/Å) of ML-CASSCF(12,10) (top left), ML-MS-CASPT2(12,10) (top right), and ML-VMC (bottom) against their respective *ab initio* reference values along the *cis*-isomerization pathway.

The MADs for a single-dissociated geometry reported in Table S10 are comparable to those observed for the geometries discussed above. Beyond the C–N distance covered by the training set (i.e., $r_{\mathrm{C-N}} > 2.25$ Å) the models enter an extrapolation regime, where the prediction accuracy deteriorates.

Despite this limitation, the ML models are able to predict the C–N bond dissociation pathway during the dynamics and yield fragment populations in very good agreement with the *ab initio* results in the case of MS-CASPT2(12,10) (see Figure S15). For CASSCF(12,10), which is more difficult for the ML model to fit as discussed above, the product fractions are

Table S10: Mean absolute deviation (MAD, kcal/mol/Å) of the forces predicted by the ML models for a single-dissociated geometry ($r_{C_1-N_1} = 2.09$ Å, $r_{C_2-N_2} = 1.44$ Å), with respect to their corresponding *ab initio* reference values. The statistical error on the last digit are indicated in parenthesis.

Method	MAD (S_0)	MAD (S_1)
VMC/CIPSI	1.27(6)	1.70(6)
CASSCF(12,10)	3.43	4.10
MS-CASPT2(12,10)	1.90	3.21

less accurate but follow the overall trend of the reference dynamics, while underestimating the total dissociation. Nevertheless, the predicted dissociation fraction remains clearly larger than those obtained with both VMC and MS-CASPT2.

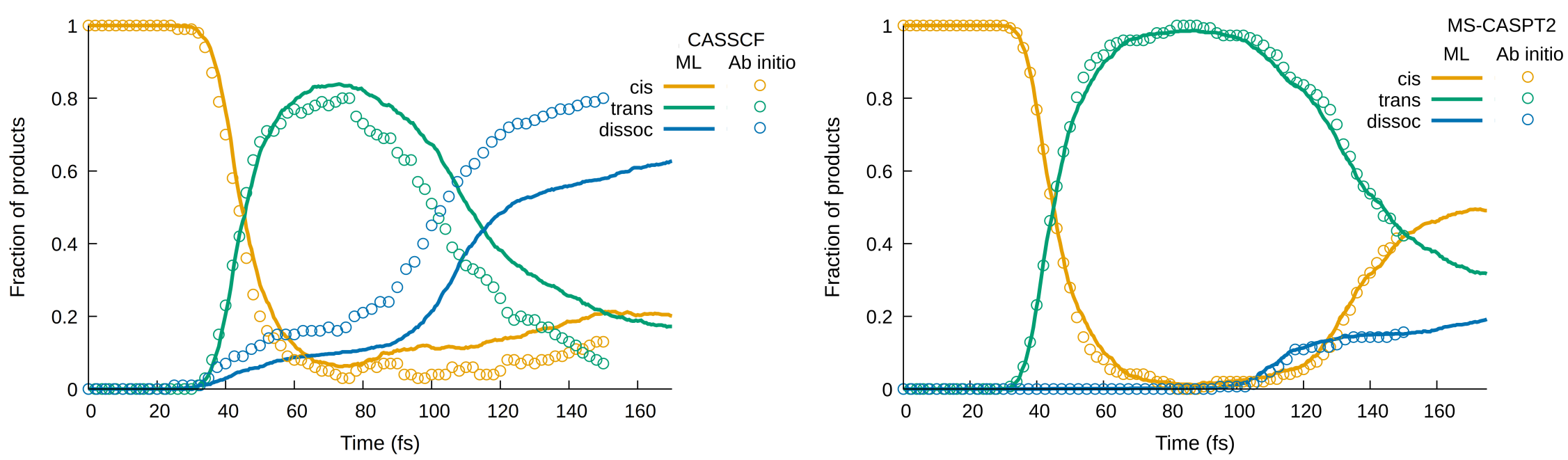

Figure S15: Time evolution of the fractions of azomethane trajectories in *cis* ($|\angle\text{CNNC}| < 90°$), *trans* ($|\angle\text{CNNC} - 180°| < 90°$), and dissociated configurations during the *cis*-isomer photodynamics. The ML-CASSCF(12,10) (left) and ML-MS-CASPT2(12,10) (right) models are compared with their respective *ab initio* reference dynamics.

S11 Force norms along the ML nonadiabatic dynamics

Figure S16 compares the time evolution of the average force RMS computed from Eq. S1 over ensembles of 1000 ML trajectories generated with the different electronic structure methods. The CASSCF force norm is consistently higher than the MS-CASPT2 and QMC values, by up to about 4 kcal/mol/Å. These larger forces may contribute to the excessive dissociation tendency observed in CASSCF dynamics.

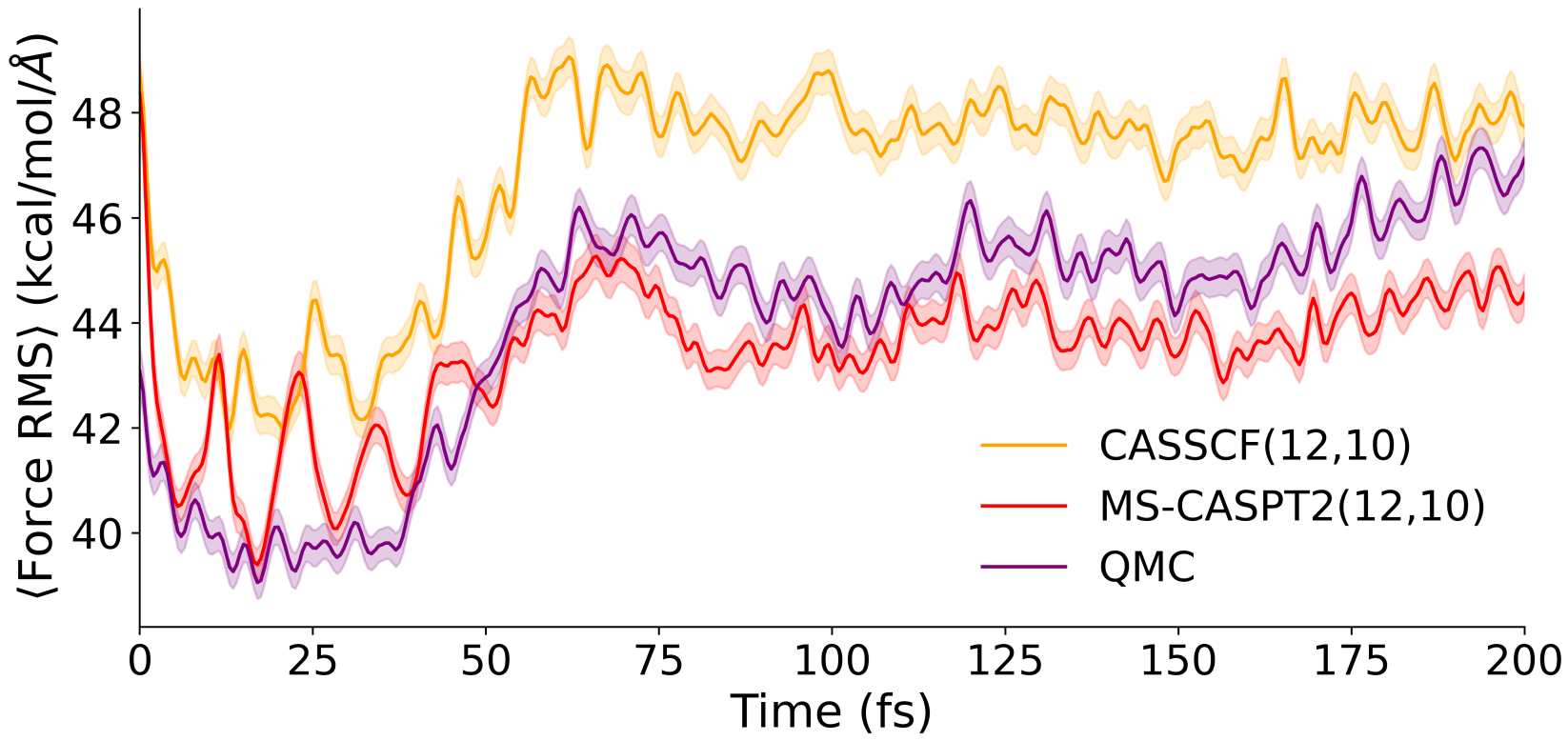

Figure S16: Time evolution of the average force RMS for the *cis*-azomethane dynamics obtained with ML models trained on data from the different electronic structure methods. Each curve is averaged over an ensemble of 1000 trajectories and the shaded regions indicate one standard deviation over the trajectory ensemble.

S12 Additional results from the ML-QMC nonadiabatic dynamics

In Figure S17, we monitor azomethane isomerization by plotting the evolution of the ∠CNNC dihedral angle for ensembles of 100 ML-VMC trajectories. In line with the VMC excited-state populations discussed in the main text, the isomerization dynamics is correctly reproduced for both *cis*- and *trans*-initiated dynamics. Specifically, some trajectories starting from the *cis* isomer pass through the strong-coupling region twice (at approximately 90° and 270°), while no recrossing occurs in the *trans*-initiated dynamics, where all trajectories hop to the ground state in the vicinity of 270°. A clear difference in the torsional timescales between the two photoinduced processes is also apparent: as also quantified in Table 2 of the main text, the onset of the first encounter with the conical-intersection region is roughly twice as late for the *trans*-initiated dynamics as for the *cis*-initiated dynamics.

The product distribution over time of the *trans*-initiated photodynamics (Figure S18) confirms that back-conversion to the *trans* isomer is negligible, consistent with the absence of a full rotation of the ∠CNNC dihedral angle. This behavior is predicted by both QMC and MS-CASPT2, which are in good agreement up to around 160 fs, when the dissociation

starts to become relevant in both cases. Similar to the *cis*-initiated photodynamics, the dissociation yield increases over time for both electronic structure methods but more rapidly in the MS-CASPT2 simulations.

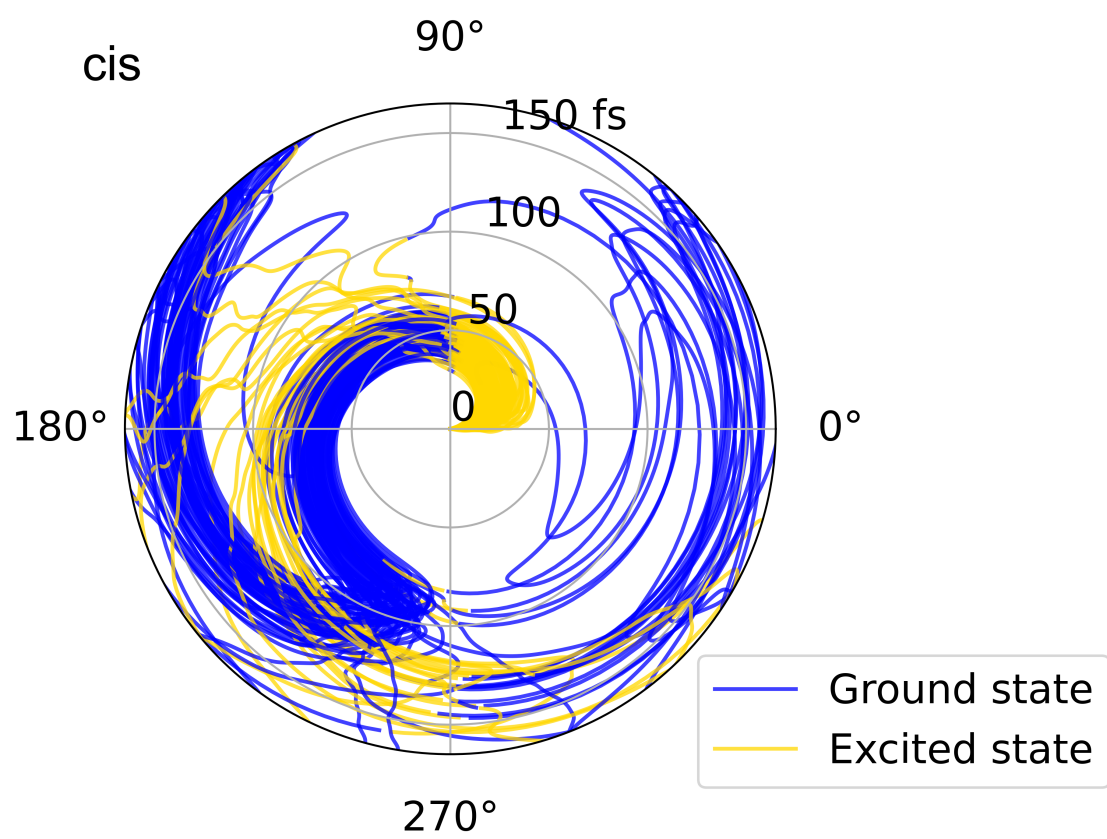

Figure S17: Polar representation of 100 ML trajectories for the *cis* (left) and *trans* (right) azomethane photodynamics computed at the VMC/CIPSI level. The angular coordinate corresponds to the ∠CNNC dihedral angle, while the radial coordinate represents the elapsed simulation time. Colors indicate the active electronic state, with the ground and excited states shown in blue and yellow, respectively.

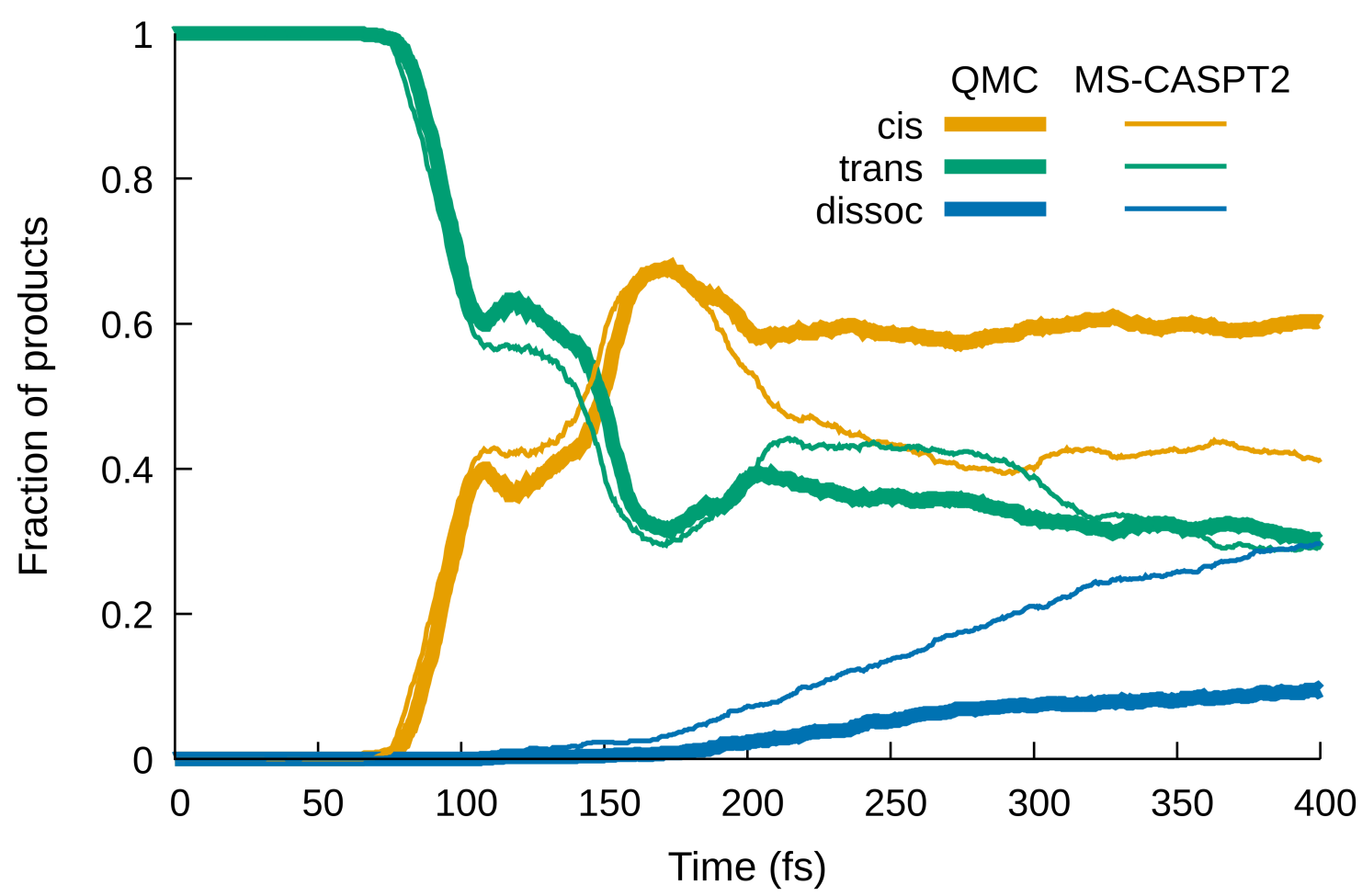

Figure S18: Fractions of azomethane trajectories in *cis* ($|\angle\mathrm{CNNC}| < 90°$), *trans* ($|\angle\mathrm{CNNC} - 180°| < 90°$), and dissociated configurations as a function of time during the *trans*-isomer photodynamics. Single and double dissociations are grouped together and identified when either or both C–N bond lengths, $r_{\mathrm{C-N}}$, exceed 2.25 Å.

References

(1) Burkatzki, M.; Filippi, C.; Dolg, M. Energy-consistent pseudopotentials for quantum Monte Carlo calculations. *J. Chem. Phys.* **2007**, *126*, 234105.

(2) Sellner, B.; Ruckenbauer, M.; Stambolić, I.; Barbatti, M.; Aquino, A. J. A.; Lischka, H. Photodynamics of Azomethane: A Nonadiabatic Surface-Hopping Study. *J. Phys. Chem. A* **2010**, *114*, 8778–8785.

(3) Hennefarth, M. R.; Truhlar, D. G.; Gagliardi, L. Semiclassical Nonadiabatic Molecular Dynamics Using Linearized Pair-Density Functional Theory. *J. Chem. Theory Comput.* **2024**, *20*, 8741–8748.

(4) Schütt, K.; Unke, O.; Gastegger, M. Equivariant message passing for the prediction of tensorial properties and molecular spectra. *Proc. Mach. Learn. Res.* **2021**, *139*, 9377–9388.

(5) Westermayr, J.; Gastegger, M.; Menger, M. F. S. J.; Mai, S.; González, L.; Marquetand, P. Machine learning enables long time scale molecular photodynamics simulations. *Chem. Sci.* **2019**, *10*, 8100–8107.

(6) Gastegger, M.; Behler, J.; Marquetand, P. Machine learning molecular dynamics for the simulation of infrared spectra. *Chem. Sci.* **2017**, *8*, 6924–6935.

(7) Dietschreit, J. C. B.; Mai, S.; González, L. The entropic barrier around the conical intersection seam. *J. Chem. Phys.* **2026**, *164*, 244112.
